# Molecular Epigenetics in the Management of Ovarian Cancer: Are We Investigating a Rational Clinical Promise?

**DOI:** 10.3389/fonc.2014.00071

**Published:** 2014-04-08

**Authors:** Ha T. Nguyen, Geng Tian, Mandi M. Murph

**Affiliations:** ^1^Department of Pharmaceutical and Biomedical Sciences, University of Georgia College of Pharmacy, Athens, GA, USA; ^2^Department of Obstetrics and Gynecology, The Second Hospital of Jilin University, Changchun, China

**Keywords:** microRNA, ovarian cancer, epigenetics, DNA methylation, histone modifications

## Abstract

Epigenetics is essentially a phenotypical change in gene expression without any alteration of the DNA sequence; the emergence of epigenetics in cancer research and mainstream oncology is fueling new hope. However, it is not yet known whether this knowledge will translate to improved clinical management of ovarian cancer. In this malignancy, women are still undergoing chemotherapy similar to what was approved in 1978, which to this day represents one of the biggest breakthroughs for treating ovarian cancer. Although liquid tumors are benefiting from epigenetically related therapies, solid tumors like ovarian cancer are not (*yet?*). Herein, we will review the science of molecular epigenetics, especially DNA methylation, histone modifications and microRNA, but also include transcription factors since they, too, are important in ovarian cancer. Pre-clinical and clinical research on the role of epigenetic modifications is also summarized. Unfortunately, ovarian cancer remains an idiopathic disease, for the most part, and there are many areas of patient management, which could benefit from improved technology. This review will also highlight the evidence suggesting that epigenetics may have pre-clinical utility in pharmacology and clinical applications for prognosis and diagnosis. Finally, drugs currently in clinical trials (i.e., histone deacetylase inhibitors) are discussed along with the promise for epigenetics in the exploitation of chemoresistance. Whether epigenetics will ultimately be the answer to better management in ovarian cancer is currently unknown; but we hope so in the future.

## Introduction to Epigenetic Modifications

**Figure 1:**
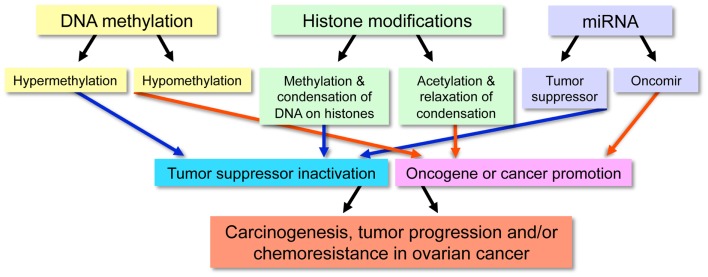
**Outline of the functional effect resulting from specific epigenetic modifications in malignancy**.

Although genetic alterations, such as gene copy-number variations, contribute to the development of cancer, classical genetics alone does not account for all acquired characteristics of cancer cells. For this reason, it is generally appreciated that epigenetic abnormalities are involved in tumorigenesis. The definition of epigenetics is the *potentially* permanent and heritable change in gene expression, which is not attributed to any alteration in the underlying DNA sequence, but results from structural adaptations and responsive outcomes on chromosome regions (1, 2). Epigenetic modifications among cancer cells result in aberrant gene expression via DNA methylation, histone modifications, and non-coding microRNAs (miRNAs) and can also include alterations among transcription factors (3), although the latter is less often emphasized in epigenetics. These modifications are associated with initiation and progression of ovarian cancers (Figure 1).

DNA methylation is the most frequently studied epigenetic phenomenon. DNA methylation occurs among cytosine residues in cytosine–guanine (CpG) dinucleotides, which are mostly distributed in the CpG-rich regions referred to as “CpG islands” (4). This type of methylation is achieved by DNA methyltransferases (DNMTs), which are a family of enzymes that serve to transfer methyl groups onto DNA (5). In humans, DNMTs are divided into two groups: DNMT1 and DNMT3.

Changes in DNA methylation regulating gene expression are widespread, appearing in both normal and cancerous cells. For example, roughly 80% of CpG dinucleotides in the human genome are subject to methylation changes throughout life. In addition, nearly 70% of all CpG islands are methylated at any given time (6). Furthermore, in normal cells, DNA methylation regulates the silenced allele of imprinted genes and also represses expression of potentially harmful DNA transposon sequences (7). Interestingly, alterations and deregulation of epigenetic events precede the transformation that generates cancer cells (8).

### Epigenetic modifications in cancer

**Figure 2:**
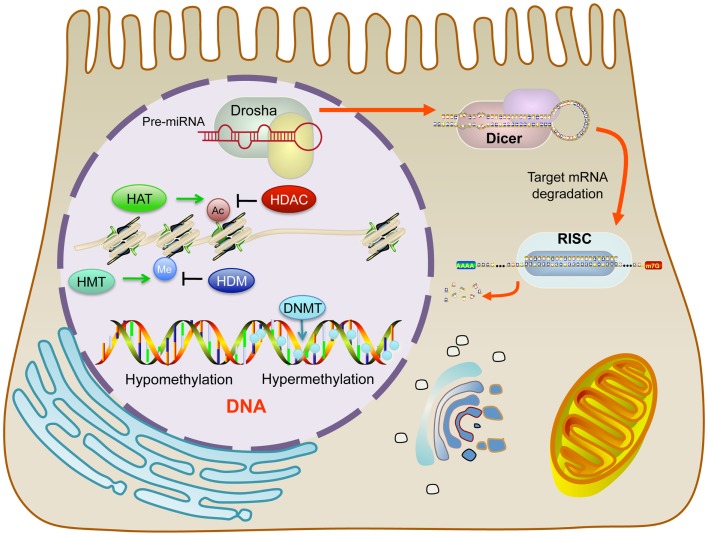
**Location of molecular epigenetic mechanisms dynamically affecting gene expression**.

Among cancer cells, DNA *hyper*-methylation is associated with gene *silencing* and DNA *hypo-*methylation with gene *expression*, both of which are widespread characteristics of malignancy (Figure 2). Most often, hypermethylated CpG islands within the DNA silence critical tumor suppressor genes, wrecking havoc on the cell’s ability to repair DNA damage, control cell growth, and inhibit proliferation. On the other hand, DNA hypo-methylation contributes to oncogenesis when previously silenced oncogenes become transcriptionally activated. In addition, DNA hypo-methylation can activate latent transposons and cause chromosomal instability in specific pericentromeric satellite regions (9–14).

Histone modifications also play important roles in epigenetic regulation. Histones are dynamic proteins that can become methylated or acetylated on specific amino acid residues, which correlates with active or repressive transcription (15, 16). An octamer of histones make up the nucleosome, which is the fundamental building-block unit of chromatin. The nucleosome contains lysine-rich histone tails extending outward from the four constituent core histone proteins (H2A, H2B, H3, and H4). These histone tails provide sites for reversible modifications to alter chromatin structure and thus, gene expression. By tightly winding and condensing chromatin or loosening up the structure of chromatin, transcription factors and other proteins are prevented or permitted access to the DNA for transcription, respectively. The target residues of histone modifications are lysine (acetylation, methylation, and ubiquitination), arginine (methylation), and serine and threonine (phosphorylation). The crosstalk between histone modifications is complicated and varied based on chromosomal domains.

Overall, the combination of histone modifications contributes largely to chromatin pattern and gene expression (17). In general, histone acetylation adds more negative charges to positive lysine, thus loosening the electrostatic interaction between histones and the DNA backbone. For this reason, the condensation of chromatin is partially regulated by histone deacetylases (HDAC), a class of deacetylating enzymes that remove acetyl groups from lysine residues of histones, ultimately causing the repression of gene expression (18, 19). If methylation also targets the same lysine residue, which means excluding acetylation, the histone methylation will have the opposite effect, compared to acetylation, and repress gene expression. However, it is not an all-encompassing rule for every single case. In fact, the situation is much more intriguing. Indeed, methylation can block repressive factors and act as a transcription-facilitating element (20).

The extent of methylation status (mono-, di-, and tri-methylation) and other types of histone modifications (phosphorylation or ubiquitination) are involved in a network of sophisticated crosstalk, determining chromatin condensation status (17). Furthermore, histone H3 phosphorylation is also suggested to interfere with the electrostatic interaction between histones and the DNA backbone, thus favoring transcription factor-induced gene expression (21). (Other types of histone modifications, such as ubiquitination and SUMOylation, are not discussed in this review.) To further convolute this process, evidence suggests that regulating gene expression may occur through crosstalk between histone modifications and DNA methylations (22–24).

MicroRNAs are small, non-coding RNAs, which are about 18–25 nucleotides in length. They negatively regulate gene expression through complementary binding to the 3′ UTR region in the promoter of targeted mRNAs, leading to mRNA degradation or translational repression, which is dependent on the level of complementarities (25, 26). Because of their unique functions, miRNAs regulate many biological changes and contribute to cancer progression. For instance, studies comparing miRNA profiles between normal and cancerous specimens identified alterations of multiple miRNA during cancer development and progression (27, 28). MicroRNAs can have dual roles in cancer progression, as tumor suppressors that repress oncogenes or as tumorigenesis factors that deregulate tumor suppressor genes (29).

### Epigenetic changes in ovarian cancer

#### Role of DNA methylation in the development of malignancy

As previously stated, DNA methylation can prevent the transcription of tumor suppressor genes. Examples of this occurrence in ovarian cancer include the *human MutL homolog 1* (*hMLH1*) and *breast cancer susceptibility gene 1* (*BRCA1*), which are two critical genes that transcribe proteins involved in the DNA damage response and DNA mismatch repair. These processes are critical in maintaining a stable chromosome and fixing damage. In ovarian cancer, the promoter regions of genes encoding these two proteins are hypermethylated, leading to the low expression levels of hMLH1 and BRCA1 (30, 31). Indeed, women with genetic mutations in *BRCA1/2* are susceptible to breast, ovarian, and (sometimes) pancreatic cancer (32) due to this aberration. Among older women with ovarian cancer, tumors are hypermethylated, leading to suppression in transforming growth factor (TGF)-beta pathway activity (33). Other silenced genes in ovarian cancer include Ras-association domain gene family 1A (RASSF1A), lost on transformation 1 (*LOT1)*, death-associated protein kinase (DAPK), target of methylation-induced silencing (*TMS1*)*/*apoptosis speck-like protein containing a CARD (*ASC*) (34–37), and insulin-like growth factor binding protein (IGFBP-3) (38). These genes encode proteins involved in regulation of the cell cycle and the promotion of apoptosis, which are important to maintain homeostasis.

#### Role of histone modifications in malignant tumorigenesis

Carcinogenesis and tumorigenesis are multifaceted; how normal tissue *precisely* undergoes stepwise changes to yield ovarian cancer and then how that progresses unregulated by mechanistic controls is largely debated. However, many aspects involved in the progression of ovarian malignancy are reported, including the role of histones in this process. For example, normal epithelial ovarian cells repress the expression of claudin-3 and claudin-4, yet these proteins are highly overexpressed in ovarian cancer. For claudin-3, this change is exclusively attributed to repressive histone marks, whereas claudin-4 repression occurs through histone modifications and DNA methylation (39). These findings explain the molecular mechanisms of repressive histone marks *likely* occurring during tumorigenesis; the rationale is that claudins are critical components of tight junctions and other claudin family members comprise gene signatures leading to worsened outcomes in ovarian cancer (40).

Another example of histone modifications affecting cell–cell interactions occurs in the TGF-beta1 receptor. This receptor is an important regulator of cell growth, cell cycle, and it also activates SMAD transcription factors. Interestingly, aberrant signaling of TGF-beta1 receptor results in histone modifications and repressive chromatin in ovarian cancer, which prevents the expression of *ADAM19*, the protein containing A Disintegrin and A Metalloprotease (41). ADAM19 is a metalloproteinase involved in cell–cell interactions and cell adhesion. Taken together, these studies suggest that histone modifications may be important epigenetic events allowing cells to alter contact with their environment.

#### MicroRNA alterations in the malignant progression of ovarian cancer

In ovarian cancer, miRNAs play a role in malignant progression. Evidence of this comes from reports that 37.1% of the miRNA genomic loci exhibit alterations in DNA copy-number (42). Other molecular mechanisms of miRNA deregulation include DNA methylation and histone modification of miRNA genes (43). Many profiling studies performed in ovarian cancer models illuminate abundant alterations. The results show that the miR-200 family and let-7 family are aberrantly regulated (Table 1) along with deregulated tumor suppressor miRNAs: miR-15a, miR-34a, and miR-34b (43, 44).

**Table 1:** **Alterations in multiple miRNAs among ovarian cancer**.

Type of cancer	Up-regulated	Down-regulated	Target	Reference
Ovarian cancer	miR-200 family (miR-200a, miR-200b, miR-200c, miR-141, miR-429), miR-214, miR-21, miR-141, miR-221, miR-146b, miR-508	miR-199a, miR-140, miR-145, miR-125b1, miR-100, let-7b, miR-125b, let-7f, miR-106b, miR-134, miR-155, miR-346, miR-424		(45–48)

In addition to genetically related explanations for miRNA deregulation, there are also changes in regulatory proteins that affect miRNA processing machinery. For example, there is a reduction in the protein expression levels of Dicer and Drosha. These two proteins are essential factors involved in the biogenesis of miRNAs (49). A loss of one dicer allele facilitates tumorigenesis while a loss of both alleles in lethal to the cell (50). Furthermore, low expression levels of Dicer and Drosha correlates with poor clinical outcomes (49).

Significant interplay is likely between miRNAs and other molecular epigenetic mechanisms of DNA methylation and histone modifications. For instance, let-7a-3 is hypermethylated in ovarian tumor samples, and the suppression of this miRNA correlates with good prognosis (51). As another example, the down-regulation of miR-101 will de-repress its target EZH2, which is a catalytic subunit of the polycomb repression complex 2 (PRC2). Because the complex tri-methylates histone H3 lysine 27, its restoration aberrantly silences multiple tumor suppression genes in cancer (45, 52, 53). MiR-140, which targets histone deacetylase 4 (HDAC4), is also reported to be down-regulated in ovarian cancer (45, 54).

## Emerging Potential of Epigenetics in the Diagnosis or Prognosis of Ovarian Cancer

### DNA methylation techniques in ovarian cancer diagnostics and prognostics

Analysis of DNA methylation status among tumor specimens is the most favored approach for developing a biomarker diagnostic/prognostic due to methylation stability, amplification ability, high-sensitivity, and relatively low cost. In fact, DNA methylation has demonstrated diagnostic and prognostic use in other types of cancers, in particular glioma (55). To detect DNA methylation, the simplest approach is to treat cells with DNA methyltransferase inhibitors (DNMTIs). The treatment will reverse the DNA methylation and result in re-expression of genes that were silenced by this mechanism. Comparison of mRNA expression levels before and after treatment will suggest candidates of methylation in cancer, which can be confirmed using additional methods. An alternative approach uses *Hpa*II, a methylation-sensitive restriction enzyme to digest genomic DNA samples prior to the amplification of digested DNA (using PCR) to compare differences in methylation levels between samples (56). In a more straightforward way, another method uses an antibody against 5-methylcytosine to precipitate methylated DNA fragments (DNA immunoprecipitation or MetDIP) (57). Collected genomic DNA fragments are then identified with array-based comparative genome hybridization to reveal human methylome maps (58).

Despite their readiness, array-based DNA methylation analysis approaches provide limited information about the extent and pattern of methylation in specific CpG regions. To overcome this drawback, bisulfite sequencing methods have been developed. Bisulfite treatment converts unmethylated cytosine residues to uracil, while methylated cytosine residues stay intact. After treatment, specific primers are used in methylation-specific PCR to amplify and help differentiate unmethylated and methylated DNA regions (59). This method is used to identify the difference in DNA methylation profiles in three major types of gynecological cancers: ovarian, endometrial, and cervical cancers (60).

To date, multiple other techniques applying bisulfite treatment have been introduced for whole-genome methylation sequencing and profiling. These include bisulfite padlock probes (BSPP), solution hybrid selection bisulfite sequencing (SHBS-seq), array capture bisulfite sequencing (ACBS-seq), and bisulfite-patch PCR (61–66); comprehensive information on methylation profiling is reviewed in Ref. (63, 67). In ovarian cancer, MethylCap-Seq for whole-genome DNA methylation profiling is a method using specific protein to capture methyl-CpG followed by high-throughput sequencing. MethylCap-Seq has been applied to analyze methylomic patterns of ovarian tumors and results suggest that hedgehog signaling pathway members (ZIC1 and ZIC4) are DNA methylation prognostic biomarkers for ovarian cancer (68, 69).

### A prospective of histone modifications in pharmacology

Although histone modifications lag behind DNA methylation in this potential application, experimental data elude to future emergence for this field. In support of this concept, research indicates that the loss of H3 histone methylation correlates with significantly reduced overall survival in ovarian cancer patients (70). In cell-based assays, proteomic techniques have been applied to profile expression-level changes, like histone modification enzymes, after treatment with a heat shock protein 90 inhibitor (HSP90). Maloney et al. suggested that similar analyses might aid pharmacology by illuminating genes and proteins involved in drug responses (71). Indeed, in ovarian cancer cells, histone de-acetylation at the RGS10-1 promoter correlates with suppression of RGS10 and chemoresistance (72). This data suggest the possibility of using histone biomarkers to determine the appropriate selection of therapeutics, particularly in cases of ovarian cancer chemoresistance, moving toward “precision” medicine in the clinic (72, 73).

The growing list of experimental techniques to examine histone modifications further alludes to emerging potential. The traditional experimental techniques available include chromatin immune precipitation (ChIP), which uses antibodies specific to acetylated histone H3 and H4 to detect histone acetylation and mass spectrometry-based proteomics to quantify histone modifications (74–78) and screen post-translational modifications among enzymes involved in epigenetic processes, like DNMT and HDAC (79). Other approaches are required to identify specific DNA sequences paired with modified histones. In this regards, the ChIP assay is coupled with a genomic tiling array (ChIP-chip) or direct sequencing (ChIP-seq). In these techniques, DNA extracted from ChIP is further processed to reveal the whole sequence, allowing a detailed mapping of histone modifications affecting the whole genome (80).

### miRNA – a biomarker for ovarian cancer?

Regarding the diagnosis of ovarian cancer among unsuspecting patients, there is no early detection biomarker that is used during routine gynecological examinations of otherwise “healthy” individuals. Although there are many candidates and extensive ongoing research for biomarkers of early stage ovarian cancer, biomarkers like CA-125 and CEA are limited to management of confirmed cases. Since it is always desirable to detect malignancy in the early stages with a minimally invasive method to collect samples, the bar is set very high for this endeavor. In addition, the accuracy requirement of a biomarker in a rare malignancy like ovarian cancer is exceedingly challenging.

In ovarian cancer, it is hypothesized that the detection of miRNAs present in circulation may be able to meet this challenge. The rationale for miRNAs as favored candidate biomarkers is due to the simplicity of obtaining blood samples and high-sensitivity detection methods. In addition, miRNAs are found in circulation within protected exosomes, which are small vesicles released into the extracellular environment from many types of cells, including tumor cells (81, 82). Interestingly, the transfer of circulating miRNAs among cells is hypothesized to be a method for internal communication within the body, similar to hormones; thus, supporting the idea of a screening approach involving miRNAs (82, 83).

There are many examples from the literature supporting the concept of miRNA biomarkers for ovarian cancer. Studies show that the expression levels of eight miRNAs have prognostic value in ovarian cancer: miR-21, miR-141, miR-200a, miR-200b, miR-200c, miR-203, miR-205, and miR-214 (45). Another study identified the expression of 21 miRNAs significantly different between ovarian cancer and normal serum specimens, including three known oncogenic miRNAs (or “oncomirs”) with biomarker potential: miR-21, miR-92, and miR-93 (84). Additionally, a large study using 300 plasma samples from ovarian cancer patients and 200 healthy controls evaluated circulating miRNAs and concluded that these are stable and specific. In this study, miR-205 and let-7f were significantly reduced in cancer specimens compared to normal controls. Moreover, let-7f has a lower expression level, which correlates with poor prognosis (85). Finally, another study suggests that among tumors, miR-9 and miR-223 deregulation is a biomarker of recurrent ovarian cancer (86).

Since miRNAs are released by cells into exosomes, studies have examined the viability of using exosomal miRNA as a potential biomarker. In this regard, research successfully used anti-epithelial cell adhesion molecule (EpCAM) to isolate exosomes secreted from ovarian tumors in plasma and compared exosome-containing miRNA expression profiles between samples from cancer patients versus healthy controls (87). Taylor and Gercel-Taylor intriguingly demonstrated that the level of tumor-derived circulating exosomes is higher in cancer patients than in normal controls. Furthermore, the level of increase correlates with higher disease grade when the exosomes are presented as concentrated protein. Finally, this study also showed that miRNAs profiles between cancer and benign specimens are significantly different (87). Because of the laboratory success in using these approaches, several groups have suggested the use of miRNAs and/or exosomes as surrogate or complementary biomarkers for biopsy profiling (85, 87, 88).

## Epigenetic Therapy in Ovarian Cancer

### Exploiting DNA methylation for therapeutic management

#### Approaches to exploit DNA methylation changes for ovarian cancer therapy

To reiterate, regions of the DNA experience changes in both hyper- and hypo-methylation during cancer initiation and/or progression. In ovarian cancer, data suggest a correlation between global and satellite DNA hypo-methylation with malignancy since an overall increase in hypo-methylation is observed among ovarian cancer tissues, in comparison with normal controls (89, 90). Furthermore, the phenomenon of satellite DNA hypo-methylation is an independent marker of poor prognosis (91).

Methylated genes are known in ovarian cancer and exhibit diagnostic potential. A study using methylation-specific PCR to screen ovarian tumor samples for six tumor suppressor genes (BRCA1, RASSF1A, APC, p14ARF, p16INK4a, and DAP-kinase) indicated that this “hyper-methylation panel” provides diagnostic information in ovarian cancer. In addition, this study further suggests that the panel is 82% sensitive and 100% specific for the detection of ovarian cancer using patient serum DNA in stage 1 (92).

The technology also holds potential use for ovarian cancer-specific prognostic information. For example, methylation-specific PCR analysis of tumor tissues from 270 patients identified that IGFBP-3 gene promoter hyper-methylation is associated with a higher risk of disease progression and mortality. Thus, hyper-methylation of IGFBP-3 is hypothesized as a biomarker for ovarian cancer outcomes, especially for patients in early stages of the disease (93).

Due to the extensive aberrant DNA methylation in cancer and the reversible nature of these events, inhibition of DNMTs is a worthy therapeutic approach to re-express tumor suppressors. DNMTIs are categorized into nucleoside and non-nucleoside analogs based on their chemical structures and mechanisms of action. DNMT nucleoside inhibitors incorporate into DNA, trap and inactivate DNMTs in the form of a covalent-DNA adduct. On the other hand, non-nucleoside DNMTIs directly block DNMT activity without covalently trapping the enzyme, thus appearing to have less toxicity (94). 5,6-Dihydro-5-azacytidine (DHAC) is a nucleoside analog of DNMTI and is in clinical trials for the treatment of ovarian cancer (95). Hydralazine, a vasodilator that treats hypertension, is also a non-nucleoside DNMTI in clinical trials for cervical cancer as both monotherapy and combination therapy (96, 97).

#### Pre-clinical studies and clinical trials exploiting DNA methylation for re-sensitization

Ovarian cancer is a treatment-intensive disease and clinics are most often re-populated by their own patients. First-line chemotherapy is initially very effective in the treatment of ovarian cancer patients, but the period of remission they achieve is often short-lived. Thus, many approaches seek to re-sensitize tumors to the previously effective drugs. In contrast, others hypothesize that because previous attempts to re-sensitize recurrent ovarian tumors to first-line therapeutics has failed, they suggest that initial combinations of compounds aimed at preventing chemoresistance is the best approach (98). However, since neither approach has achieved bona-fide, proof-of-principle, research into *both* approaches is ongoing.

Researchers are evaluating the application of DNA methylation for chemotherapy re-sensitization. For example, the hyper-methylation of hMLH1 (human MutL homolog 1) inhibits the apoptotic response to platinum-DNA abduct formation from platinum chemotherapy. Thus, this hyper-methylation is considered a major molecular cause of acquired resistance to platinum chemotherapy in ovarian cancer (99). In addition, the presence of methylated hMLH1 DNA in plasma after chemotherapy predicts poor survival for ovarian cancer patients (100). Interestingly, the hMLH1 activity is restored after treatment with 5-aza-2′-deoxycytidine (decitabine) and so is the re-sensitization of ovarian cancer to cisplatin (101).

Another example of DNA methylation and chemotherapy re-sensitization surrounds RAS-associated domain family protein 1a (RASSF1A). The promoter methylation of RASSF1A is highly associated with ovarian cancer (34). RASSF1A binds to tubulin and promotes microtubule polymerization and stabilization (102, 103). The presence of RASSF1A blocks genome instability induced by RAS (85, 104). RASSF1A also causes cell cycle arrest through blocking Cyclin D1 accumulation (105). For all these reasons, RASSF1A is an interesting target for restoration.

Many pre-clinical studies present evidence that DNMT inhibitors are efficient in de-repressing tumor suppressor genes. This intimates that DNMT inhibitors may have therapeutic potential in combination regimens to overcome resistance and/or provide synergistic effects (106, 107). For example, decitabine re-sensitizes chemoresistant ovarian tumor xenografts to cisplatin, carboplatin, temozolomide, and epirubicin (101). Restoration of RASFF1A by inhibiting DNMT also increases ovarian cancer cell sensitivity to paclitaxel (108).

Indeed, DNMT inhibitors are also showing some success in the clinic (Table 2). Decitabine is undergoing clinical trials with carboplatin for patients with recurrent, platinum-resistant ovarian cancer (109). A report of a phase II clinical trial of low-dose decitabine combined with carboplatin for heavily treated and platinum-resistant ovarian cancer patients showed positive results. Low-dose decitabine altered the methylation of genes in tumorigenesis pathways, including the demethylation of hMLH1, RASSF1A, HOXA10, and HOXA11, leading to re-sensitization to carboplatin, increased response rate, and prolonged progression-free survival (110).

**Table 2:** **Epigenetic drugs in gynecological cancer trials**.

Drugs	Other names	Group	Types of diseases
Valproic acid		HDAC inhibitors	Cervical, ovarian cancers
Belinostat		HDAC inhibitors	Gynecological cancers
Decitabine		DNMT inhibitors	Ovarian cancer
Hydralazine		DNMT inhibitors	Cervical cancer
Dihydro-5-azacytidine	DHAC	DNMT inhibitors	Ovarian cancer

Hypo-methylation treatment, on the other hand, due to its non-specific effects, can be detrimental. One known example is the Fanconi anemia (FANC)–BRCA pathway in ovarian cancer. The malfunction of genes in FANC pathway leads to devastating mutagen hypersensitivity (111). In cancer treatment, the FNAC–BRCA pathway plays a critical role in the response of cells to DNA-crosslinking agents. However, it was observed that, in ovarian cancer, FANC is inactivated due to hyper-methylation, and the demethylation of FANC is associated with ovarian tumor progression and acquired cisplatin resistance (112). In addition, there are oncogenic genes overexpressed by hypo-methylation in ovarian cancer, such as synuclein-γ and mammalian heparanase (endo-beta-glucuronidase) (113–115).

### Histone modifications: Histone deacetylase inhibition in clinical trials

Histone deacetylases are enzymes that remove acetyl groups and have long been studied for treatment of cancer, in general, as well as of gynecological cancers, specifically. Although HDAC overexpression occurs in many types of cancers (116, 117), siRNA silencing HDAC1 and HDAC2 inhibits growth and promotes apoptosis in ovarian and cervical cancer cells (118, 119). Similarly, HDAC6 facilitates oncogenic transformation in ovarian cancer (120). Thus, there is sufficient support for the rational targeting and inhibiting HDAC within the treatment of this malignancy.

Based on their chemical structures, HDAC inhibitors are divided into four majors groups: short-chain fatty acid, hydroxamic acid, cyclic tetrapeptide, and benzamide (121). For example, valproic acid, a reagent belonging to the short-chain fatty acid group (also known as an anti-epileptic and mood stabilizer) is in clinical trials for the treatment of cervical and ovarian cancers (122–124). Scriptaid, another HDAC inhibitor in the hydroxamic group, showed growth inhibition and apoptosis-inducing potential in ovarian and endometrial cancers (125). Apicidin, an HDAC inhibitor in the cyclic tetrapeptide group, is also studied for its anti-growth effects in ovarian and endometrial cancer cells (126).

The aberrant expression of HDACs in gynecological cancers is likely associated with *de novo* resistance and/or poor chemotherapeutic efficacy and thus, chemoresistance development. As with nearly all new drugs, HDAC inhibitors are proposed for combination therapy to strengthen therapeutic efficacy as well as to minimize chemoresistance. Valproic acid has been studied in combination with several cytotoxic drugs, such as methotrexate or epirubicin, for synergistic or antagonistic effects in other types of cancer (127, 128). Belinostat (PDX101), a novel HDAC inhibitor in the hydroxamic acid group, displayed anticancer effects as a single agent as well as in combination by increasing the acetylation of tubulin induced by docetaxel and the phosphorylation of H2AX induced by carboplatin (129). Belinostat is under phase II clinical trials for gynecological cancer treatment in combination with platinum or paclitaxel to enhance effectiveness and help overcome resistance (130–134). OSU-HDAC42 (or AR-42), another new short-chain fatty acid HDAC inhibitor, has anti-growth effects on ovarian cancer cells but not normal epithelial cells. The compound re-sensitizes platinum-resisted ovarian tumors *in vivo* to cisplatin and may have great potential for combinations with platinum agents (135).

### Exploiting miRNAs for re-sensitization of chemoresistant disease

The goal of targeting miRNAs in cancer treatment is to down-regulate oncomirs, to inhibit mRNAs that will become oncogenic proteins, or to restore tumor suppressor miRNAs. Multiple techniques have been developed to target oncomirs, such as locked nucleic acid (LNA), miRNA sponges, miRNA masking, or small-molecule inhibitors (136–139). On the other hand, the most straightforward way to restore tumor suppressor miRNAs is to deliver pre-miRNA precursors or miRNA mimics. However, straightforward it may appear, it is the targeted *delivery* of these molecules that represents a major obstacle.

A critical clinical problem in ovarian cancer is chemoresistance. Multiple studies in the field have focused on the roles of miRNAs in overcoming resistance to chemotherapeutic agents. Many miRNAs are reported as expressed differently between chemosensitive and chemoresistant ovarian cell lines, such as miR-30c, miR-130a, miR-335, among those, miR-130a is confirmed to target resistant factor M-CSF (Table 3) (140). In addition, the enforced expression of miR-30c-2-3p into chemoresistant and chemo-insensitive ovarian cancer cells significantly reduces their viability, independently of cisplatin or paclitaxel, without affecting immortalized ovarian surface epithelial cells (141).

**Table 3:** **miRNAs involved in chemoresistance**.

miRNAs	Trend in resistance	Target genes	Resisted drugs	References
miR-200 family		B-tubulin III TGF-beta2, ZEB1	Paclitaxel	(142, 143)
Let-7i	Reduced		Cisplatin	(144)
miR-30c, miR-130a, miR-335	Reduced	M-CSF (target of miR-130a)	Paclitaxel, cisplatin	(140)
miR-214	Increased	PTEN	Cisplatin	(46)
miR-27a, miR-21, miR-451	Increased	MDR1 (indirectly through HIPK2, in case of miR-27a)	Paclitaxel	
Let-7g	Reduced	MDR1 (indirectly through IMP-1)	Taxane agents	(145)
miR-27a, miR-23a, miR-30c, let-7g, miR-199a-3p, miR-378, miR-625	Increased		Platinum agents	(146)
Let-7a		Caspase-3	Paclitaxel	(147, 148)
miR-130b	Decreased	CSF-1	Cisplatin, paclitaxel	(149)
miR-141	Increased	KEAP1	Cisplatin	(150)
miR-106a, miR-591	Increased (miR-106a)	BCL-10, caspase-7, ZEB1	Paclitaxel	(151)
	Decreased (miR-591)	
miR-29	Decreased	COL1A1	Cisplatin	(152)
miR-182	Increased	PDCD4 TCEAL7	Paclitaxel	(153, 154)

Although the miR-200 family is a potential prognostic factor of ovarian and endometrial cancer (87, 155), it may have a role in re-sensitization. The low expression of miR-200c in cancer leads to an increase in the expression of its target, class III β-tubulin (TUBB3) (142). Since the expression of TUBB3 is required for chemoresistance to microtubule-binding agents (e.g., taxanes and vinca alkaloids), restoration of miR-200c down-regulates TUBB3, and effectively re-sensitizes ovarian cancer cells to paclitaxel (142, 143, 156).

In addition to the miR-200 family, several members of the let-7 family are well documented as down-regulated in ovarian cancer, including let-7a, let-7b, let-7c, let-7d, and let-7i (44–48, 144). Among these, let-7a is a potential biomarker for the selection of chemotherapy in ovarian cancer. Patients with low let-7 showed good response using platinum-paclitaxel combination therapy, while patients with higher let-7a had better survival using platinum without paclitaxel; adding paclitaxel to this group produced worse progression-free and overall survival (147). The down-regulation of another member of the let-7 family, let-7i, is associated with resistance of ovarian cancer cells to cisplatin, which suggests that let-7i could be used as a therapeutic target to overcome platinum resistance and as a biomarker to predict chemotherapy response in ovarian cancer patients (144). Another study observed that the let-7 family member, let-7g, down-regulates the *multiple drug resistance 1* (*MDR1*) gene, one of the major factors causing paclitaxel resistance in ovarian cancer (145).

There are numerous other miRNAs that have roles in ovarian cancer chemoresistance with known mechanisms. These include, but are not limited to, miRNAs like miR-214, miR-27a, and miR-451. MiR-214 targets PTEN, a known tumor suppressor, therefore, inducing cell survival and cisplatin resistance (46). MiR-27a increases MDR1/P-glycoprotein expression in ovarian cancer cells by targeting HIPK2 as an intermediate (157). Similarly, miR-451 and miR-21 also facilitate MDR1/P-glycoprotein overexpression, leading to paclitaxel resistance in ovarian cancer cells (158, 159).

### Targeting transcription factors in ovarian cancer

Cancer is often a condition with aberrant gene expression, specifically involving the overexpression of oncogenes. Altered transcription factors are recognized as an epigenetic entity comprising the “ovarian cancer cell epigenome” (3). This is not surprising given the relationship between transcription factors and structural (not sequence) alterations of the DNA (via DNA methylation and histone modifications).

There are numerous examples of aberrant transcription factors in cancer. Perhaps the most prominent of all is the tumor suppressor protein p53. Mutations of *TP53*, the gene encoding p53, are very common in ovarian cancer (160). In fact, nearly 100% of patients with high-grade serous epithelial ovarian cancer have mutations in p53. Overall, at least 50% of all ovarian tumors have mutations in p53, most of which are point mutations leading to amino acid substitutions. These are detrimental to the p53 protein because they affect the DNA-binding domain of the transcription factor (161). Unfortunately, therapeutic intervention using p53 as the target molecule has not yet achieved measurable success (98).

The transcription factor and tumor suppressor protein p53 are critical to the signaling pathways of cell cycle arrest and apoptosis. Once activated by DNA damage detection or UV radiation, p53 induces the expression of many well-known apoptosis inducers and other tumor suppressors, such as p21^Cip1^, BAX, PTEN, and TSP-1. Because of this important role, the inactivation of p53 facilitates many phases of tumor progression as DNA damage cannot be repaired and apoptotic pathways cannot be activated when necessary (161).

Beside p53, other transcription factors have important roles in ovarian cancer pathology. For example, Gil1 (glioma-associated oncogene homolog 1) expression is elevated in advanced serous ovarian cancer and this event is correlated with unfavorable survival (162). Since transcription factor alterations can have a tremendous impact on the balance of the entire biological system, targeting transcription factors is an emerging trend in cancer therapy research. The possibility of exerting broad control over the system could be a powerful method of regaining regulatory control. This is especially in light of lessons learned in other cancers whereby targeting one particular kinase or protein in a larger signaling pathway leads to the rapid acquisition of therapeutic resistance.

On the other hand, inhibiting particular transcription factors could provide specificity toward malignant overexpression events in cancer (e.g., oncogenes, oncomirs, etc.). Furthermore, this approach is appealing because it might produce more tolerance among healthy cells due to redundancies in normal signaling pathways. Two major approaches in targeting transcription factors are post-transcriptional silencing (using siRNAs or miRNAs) or blocking the binding of transcription factors to DNA during activation. Another indirect approach is regulating histone-modified enzymes and DNA methyltransferase if the target transcription factor is mis-regulated through histone modification and/or DNA methylation.

Many well-known transcription factors are studied as potential targets in general cancer treatment, such as STATs, NF-κB, and Notch1 (163–165). In gynecological cancers, multiple studies have reported the involvement of transcription factors in cancer progression and described them as potential targets for cancer treatment. In ovarian cancer, the blockage of STAT3 using a decoy oligodeoxynucleotide inhibits cancer cell growth (166). Another study in ovarian cancer also showed that suppression of NF-κB activity using minocycline, a tetracycline, had beneficial effects both *in vitro* and *in vivo* (167). More research is needed in this area to refine this approach and evaluate its worthiness.

## Conclusion: Epigenetic Therapy

By undertaking research projects focused on epigenetic-related translational applications, are basic scientists investigating a rational clinical promise? To address this question, it is necessary to review the successful progression of ideas from the laboratory into clinic therapeutics. Although no epigenetic drugs have advanced into the clinic for use against ovarian cancer, there are several FDA-approved therapeutics (e.g., vorinostat, decitabine, and romidepsin) for other types of cancer, especially liquid tumors. Clinical trials are ongoing for ovarian cancer with epigenetic therapeutics (Table 2). Since first-line therapy often results in disease remission, predictions support using new drugs in combination therapy. Although hope lingers for PARP inhibitors, this class of drugs may only treat a specific population of women (168). Whether using epigenetic modifiers will achieve significant improvements in overall survival is incalculable. Nevertheless, to advance patient outcomes in ovarian cancer, new approaches are required – the previous *breakthroughs* occurred in 1978 (cisplatin) and 1992 (paclitaxel). An improved therapeutic regimen for ovarian cancer is long overdue. Epigenetics provide hope in a new direction.

## Conflict of Interest Statement

The authors declare that the research was conducted in the absence of any commercial or financial relationships that could be construed as a potential conflict of interest.

## References

[1] Goldberg AD Allis CD Bernstein E Epigenetics: a landscape takes shape. Cell (2007) 128(4):635–8 10.1016/j.cell.2007.02.006 17320500

[2] Bird A Perceptions of epigenetics. Nature (2007) 447(7143):396–8 10.1038/nature05913 17522671

[3] Balch C Fang F Matei DE Huang TH Nephew KP Minireview: epigenetic changes in ovarian cancer. Endocrinology (2009) 150(9):4003–11 10.1210/en.2009-0404 19574400 PMC2736079

[4] Iacobuzio-Donahue CA Epigenetic changes in cancer. Annu Rev Pathol Mech Dis (2009) 4:229–49 10.1146/annurev.pathol.3.121806.151442 18840073

[5] Robertson KD Jones PA DNA methylation: past, present and future directions. Carcinogenesis (2000) 21(3):461–7 10.1093/carcin/21.3.461 10688866

[6] Zhu X Lee K Asa SL Ezzat S Epigenetic silencing through DNA and histone methylation of fibroblast growth factor receptor 2 in neoplastic pituitary cells. Am J Pathol (2007) 170(5):1618–28 10.2353/ajpath.2007.061111 17456767 PMC1854956

[7] Bestor TH The host defence function of genomic methylation patterns. Novartis Found Symp (1998) 214:187–95; discussion 195–9, 228–32. 10.1002/9780470515501.ch11 9601018

[8] Rodriguez-Paredes M Esteller M Cancer epigenetics reaches mainstream oncology. Nat Med (2011) 17(3):330–9 10.1038/nm.2305 21386836

[9] Alves G Tatro A Fanning T Differential methylation of human LINE-1 retrotransposons in malignant cells. Gene (1996) 176(1–2):39–44 10.1016/0378-1119(96)00205-3 8918229

[10] Costello JF Plass C Methylation matters. J Med Genet (2001) 38(5):285–303 10.1136/jmg.38.5.285 11333864 PMC1734882

[11] Eden A Gaudet F Waghmare A Jaenisch R Chromosomal instability and tumors promoted by DNA hypomethylation. Science (2003) 300(5618):455 10.1126/science.1083557 12702868

[12] Gaudet F Hodgson JG Eden A Jackson-Grusby L Dausman J Gray JW Induction of tumors in mice by genomic hypomethylation. Science (2003) 300(5618):489–92 10.1126/science.1083558 12702876

[13] Tuck-Muller CM Narayan A Tsien F Smeets DF Sawyer J Fiala ES DNA hypomethylation and unusual chromosome instability in cell lines from ICF syndrome patients. Cytogenet Cell Genet (2000) 89(1–2):121–8 10.1159/000015590 10894953

[14] Kanai Y Genome-wide DNA methylation profiles in precancerous conditions and cancers. Cancer Sci (2010) 101(1):36–45 10.1111/j.1349-7006.2009.01383.x 19891661 PMC11159222

[15] Esteller M Cancer epigenomics: DNA methylomes and histone-modification maps. Nat Rev Genet (2007) 8(4):286–98 10.1038/nrg2005 17339880

[16] Kouzarides T Chromatin modifications and their function. Cell (2007) 128(4):693–705 10.1016/j.cell.2007.02.005 17320507

[17] Fischle W Wang Y Allis CD Histone and chromatin cross-talk. Curr Opin Cell Biol (2003) 15(2):172–83 10.1016/S0955-0674(03)00013-9 12648673

[18] Roth SY Denu JM Allis CD Histone acetyltransferases. Annu Rev Biochem (2001) 70:81–120 10.1146/annurev.biochem.70.1.81 11395403

[19] Thiagalingam S Cheng KH Lee HJ Mineva N Thiagalingam A Ponte JF Histone deacetylases: unique players in shaping the epigenetic histone code. Ann N Y Acad Sci (2003) 983:84–100 10.1111/j.1749-6632.2003.tb05964.x 12724214

[20] Beisel C Imhof A Greene J Kremmer E Sauer F Histone methylation by the Drosophila epigenetic transcriptional regulator Ash1. Nature (2002) 419(6909):857–62 10.1038/nature01126 12397363

[21] Cheung P Allis CD Sassone-Corsi P Signaling to chromatin through histone modifications. Cell (2000) 103(2):263–71 10.1016/S0092-8674(00)00118-5 11057899

[22] Fuks F DNA methylation and histone modifications: teaming up to silence genes. Curr Opin Genet Dev (2005) 15(5):490–5 10.1016/j.gde.2005.08.002 16098738

[23] Simon JA Lange CA Roles of the EZH2 histone methyltransferase in cancer epigenetics. Mutat Res (2008) 647(1–2):21–9 10.1016/j.mrfmmm.2008.07.010 18723033

[24] Vaissiere T Sawan C Herceg Z Epigenetic interplay between histone modifications and DNA methylation in gene silencing. Mutat Res (2008) 659(1–2):40–8 10.1016/j.mrrev.2008.02.004 18407786

[25] Baek D Villén J Shin C Camargo FD Gygi SP Bartel DP The impact of microRNAs on protein output. Nature (2008) 455(7209):64–71 10.1038/nature07242 18668037 PMC2745094

[26] Bartel DP MicroRNAs: genomics, biogenesis, mechanism, and function. Cell (2004) 116(2):281–97 10.1016/S0092-8674(04)00045-5 14744438

[27] Calin GA Croce CM MicroRNA signatures in human cancers. Nat Rev Cancer (2006) 6(11):857–66 10.1038/nrc1997 17060945

[28] Lu J Getz G Miska EA Alvarez-Saavedra E Lamb J Peck D MicroRNA expression profiles classify human cancers. Nature (2005) 435(7043):834–8 10.1038/nature03702 15944708

[29] Esquela-Kerscher A Slack FJ Oncomirs – microRNAs with a role in cancer. Nat Rev Cancer (2006) 6(4):259–69 10.1038/nrc1840 16557279

[30] Bi FF Li D Cao C Li CY Yang Q Regulation of angiotensin II type 1 receptor expression in ovarian cancer: a potential role for BRCA1. J Ovarian Res (2013) 6(1):89 10.1186/1757-2215-6-89 24321324 PMC4029559

[31] Meng CF Su B Li W DNA demethylation is superior to histone acetylation for reactivating cancer-associated genes in ovarian cancer cells. Mol Med Rep (2011) 4(6):1273–8 10.3892/mmr.2011.557 21850374

[32] Ferrone CR Levine DA Tang LH Allen PJ Jarnagin W Brennan MF BRCA germline mutations in Jewish patients with pancreatic adenocarcinoma. J Clin Oncol (2009) 27(3):433–8 10.1200/JCO.2008.18.5546 19064968 PMC3657622

[33] Matsumura N Huang Z Mori S Baba T Fujii S Konishi I Epigenetic suppression of the TGF-beta pathway revealed by transcriptome profiling in ovarian cancer. Genome Res (2011) 21(1):74–82 10.1101/gr.108803.110 21156726 PMC3012928

[34] Shi H Li Y Wang X Lu C Yang L Gu C Association between RASSF1A promoter methylation and ovarian cancer: a meta-analysis. PLoS One (2013) 8(10):e76787 10.1371/journal.pone.0076787 24116157 PMC3792894

[35] Abdollahi A Pisarcik D Roberts D Weinstein J Cairns P Hamilton TC LOT1 (PLAGL1/ZAC1), the candidate tumor suppressor gene at chromosome 6q24-25, is epigenetically regulated in cancer. J Biol Chem (2003) 278(8):6041–9 10.1074/jbc.M210361200 12473647

[36] Collins Y Dicioccio R Keitz B Lele S Odunsi K Methylation of death-associated protein kinase in ovarian carcinomas. Int J Gynecol Cancer (2006) 16(Suppl 1):195–9 10.1111/j.1525-1438.2006.00506.x 16515590

[37] Terasawa K Sagae S Toyota M Tsukada K Ogi K Satoh A Epigenetic inactivation of TMS1/ASC in ovarian cancer. Clin Cancer Res (2004) 10(6):2000–6 10.1158/1078-0432.CCR-0932-03 15041718

[38] Torng PL Lin CW Chan MW Yang HW Huang SC Lin CT Promoter methylation of IGFBP-3 and p53 expression in ovarian endometrioid carcinoma. Mol Cancer (2009) 8:120 10.1186/1476-4598-8-120 20003326 PMC2799391

[39] Kwon MJ Kim SS Choi YL Jung HS Balch C Kim SH Derepression of CLDN3 and CLDN4 during ovarian tumorigenesis is associated with loss of repressive histone modifications. Carcinogenesis (2010) 31(6):974–83 10.1093/carcin/bgp336 20053926 PMC2878357

[40] Murph MM Liu W Yu S Lu Y Hall H Hennessy BT Lysophosphatidic acid-induced transcriptional profile represents serous epithelial ovarian carcinoma and worsened prognosis. PLoS One (2009) 4(5):e5583 10.1371/journal.pone.0005583 19440550 PMC2679144

[41] Chan MW Huang YW Hartman-Frey C Kuo CT Deatherage D Qin H Aberrant transforming growth factor beta1 signaling and SMAD4 nuclear translocation confer epigenetic repression of ADAM19 in ovarian cancer. Neoplasia (2008) 10(9):908–19 10.1593/neo.08540 18714391 PMC2517635

[42] Zhang L Huang J Yang N Greshock J Megraw MS Giannakakis A microRNAs exhibit high frequency genomic alterations in human cancer. Proc Natl Acad Sci U S A (2006) 103(24):9136–41 10.1073/pnas.0508889103 16754881 PMC1474008

[43] Gilabert-Estelles J Braza-Boils A Ramon LA Zorio E Medina P Espana F Role of microRNAs in gynecological pathology. Curr Med Chem (2012) 19(15):2406–13 10.2174/092986712800269362 22455593

[44] Zhang L Volinia S Bonome T Calin GA Greshock J Yang N Genomic and epigenetic alterations deregulate microRNA expression in human epithelial ovarian cancer. Proc Natl Acad Sci U S A (2008) 105(19):7004–9 10.1073/pnas.0801615105 18458333 PMC2383982

[45] Iorio MV Visone R Di Leva G Donati V Petrocca F Casalini P MicroRNA signatures in human ovarian cancer. Cancer Res (2007) 67(18):8699–707 10.1158/0008-5472.CAN-07-1936 17875710

[46] Yang H Kong W He L Zhao JJ O’Donnell JD Wang J MicroRNA expression profiling in human ovarian cancer: miR-214 induces cell survival and cisplatin resistance by targeting PTEN. Cancer Res (2008) 68(2):425–33 10.1158/0008-5472.CAN-07-2488 18199536

[47] Nam EJ Yoon H Kim SW Kim H Kim YT Kim JH MicroRNA expression profiles in serous ovarian carcinoma. Clin Cancer Res (2008) 14(9):2690–5 10.1158/1078-0432.CCR-07-1731 18451233

[48] Dahiya N Sherman-Baust CA Wang TL Davidson B Shih IEM Zhang Y MicroRNA expression and identification of putative miRNA targets in ovarian cancer. PLoS One (2008) 3(6):e2436 10.1371/journal.pone.0002436 18560586 PMC2410296

[49] Merritt WM Lin YG Han LY Kamat AA Spannuth WA Schmandt R Dicer, Drosha, and outcomes in patients with ovarian cancer. N Engl J Med (2008) 359(25):2641–50 10.1056/NEJMoa0803785 19092150 PMC2710981

[50] Kumar MS Pester RE Chen CY Lane K Chin C Lu J Dicer1 functions as a haploinsufficient tumor suppressor. Genes Dev (2009) 23(23):2700–4 10.1101/gad.1848209 19903759 PMC2788328

[51] Lu L Katsaros D de laLongrais IA Sochirca O Yu H Hypermethylation of let-7a-3 in epithelial ovarian cancer is associated with low insulin-like growth factor-II expression and favorable prognosis. Cancer Res (2007) 67(21):10117–22 10.1158/0008-5472.CAN-07-2544 17974952

[52] Friedman JM Liang G Liu CC Wolff EM Tsai YC Ye W The putative tumor suppressor microRNA-101 modulates the cancer epigenome by repressing the polycomb group protein EZH2. Cancer Res (2009) 69(6):2623–9 10.1158/0008-5472.CAN-08-3114 19258506

[53] Iorio MV Piovan C Croce CM Interplay between microRNAs and the epigenetic machinery: an intricate network. Biochim Biophys Acta (2010) 1799(10–12):694–701 10.1016/j.bbagrm.2010.05.005 20493980

[54] Tuddenham L Wheeler G Ntounia-Fousara S Waters J Hajihosseini MK Clark I The cartilage specific microRNA-140 targets histone deacetylase 4 in mouse cells. FEBS Lett (2006) 580(17):4214–7 10.1016/j.febslet.2006.06.080 16828749

[55] Esteller M Epigenetics in cancer. N Engl J Med (2008) 358(11):1148–59 10.1056/NEJMra072067 18337604

[56] Ushijima T Morimura K Hosoya Y Okonogi H Tatematsu M Sugimura T Establishment of methylation-sensitive-representational difference analysis and isolation of hypo- and hypermethylated genomic fragments in mouse liver tumors. Proc Natl Acad Sci U S A (1997) 94(6):2284–9 10.1073/pnas.94.6.2284 9122186 PMC20079

[57] Weber M Davies JJ Wittig D Oakeley EJ Haase M Lam WL Chromosome-wide and promoter-specific analyses identify sites of differential DNA methylation in normal and transformed human cells. Nat Genet (2005) 37(8):853–62 10.1038/ng1598 16007088

[58] Wilson IM Davies JJ Weber M Brown CJ Alvarez CE MacAulay C Epigenomics: mapping the methylome. Cell Cycle (2006) 5(2):155–8 10.4161/cc.5.2.2367 16397413

[59] Herman JG Graff JR Myöhänen S Nelkin BD Baylin SB Methylation-specific PCR: a novel PCR assay for methylation status of CpG islands. Proc Natl Acad Sci U S A (1996) 93(18):9821–6 10.1073/pnas.93.18.9821 8790415 PMC38513

[60] Yang HJ Liu VW Wang Y Tsang PC Ngan HY Differential DNA methylation profiles in gynecological cancers and correlation with clinico-pathological data. BMC Cancer (2006) 6:212 10.1186/1471-2407-6-212 16928264 PMC1560388

[61] Hodges E Xuan Z Balija V Kramer M Molla MN Smith SW Genome-wide in situ exon capture for selective resequencing. Nat Genet (2007) 39(12):1522–7 10.1038/ng.2007.42 17982454

[62] Porreca GJ Zhang K Li JB Xie B Austin D Vassallo SL Multiplex amplification of large sets of human exons. Nat Methods (2007) 4(11):931–6 10.1038/nmeth1110 17934468

[63] Lee EJ Pei L Srivastava G Joshi T Kushwaha G Choi JH Targeted bisulfite sequencing by solution hybrid selection and massively parallel sequencing. Nucleic Acids Res (2011) 39(19):e127 10.1093/nar/gkr598 21785137 PMC3201883

[64] Hodges E Smith AD Kendall J Xuan Z Ravi K Rooks M High definition profiling of mammalian DNA methylation by array capture and single molecule bisulfite sequencing. Genome Res (2009) 19(9):1593–605 10.1101/gr.095190.109 19581485 PMC2752124

[65] Varley KE Mitra RD Bisulfite patch PCR enables multiplexed sequencing of promoter methylation across cancer samples. Genome Res (2010) 20(9):1279–87 10.1101/gr.101212.109 20627893 PMC2928506

[66] Lee EJ Luo J Wilson JM Shi H Analyzing the cancer methylome through targeted bisulfite sequencing. Cancer Lett (2013) 340(2):171–8 10.1016/j.canlet.2012.10.040 23200671 PMC3616138

[67] Shanmuganathan R Basheer NB Amirthalingam L Muthukumar H Kaliaperumal R Shanmugam K Conventional and nanotechniques for DNA methylation profiling. J Mol Diagn (2013) 15(1):17–26 10.1016/j.jmoldx.2012.06.007 23127612

[68] Brinkman AB Simmer F Ma K Kaan A Zhu J Stunnenberg HG Whole-genome DNA methylation profiling using MethylCap-seq. Methods (2010) 52(3):232–6 10.1016/j.ymeth.2010.06.012 20542119

[69] Huang RL Gu F Kirma NB Ruan J Chen CL Wang HC Comprehensive methylome analysis of ovarian tumors reveals hedgehog signaling pathway regulators as prognostic DNA methylation biomarkers. Epigenetics (2013) 8(6):624–34 10.4161/epi.24816 23774800 PMC3857342

[70] Wei Y Xia W Zhang Z Liu J Wang H Adsay NV Loss of trimethylation at lysine 27 of histone H3 is a predictor of poor outcome in breast, ovarian, and pancreatic cancers. Mol Carcinog (2008) 47(9):701–6 10.1002/mc.20413 18176935 PMC2580832

[71] Maloney A Clarke PA Naaby-Hansen S Stein R Koopman JO Akpan A Gene and protein expression profiling of human ovarian cancer cells treated with the heat shock protein 90 inhibitor 17-allylamino-17-demethoxygeldanamycin. Cancer Res (2007) 67(7):3239–53 10.1158/0008-5472.CAN-06-2968 17409432

[72] Ali MW Cacan E Liu Y Pierce JY Creasman WT Murph MM Transcriptional suppression, DNA methylation, and histone deacetylation of the regulator of G-protein signaling 10 (RGS10) gene in ovarian cancer cells. PLoS One (2013) 8(3):e60185 10.1371/journal.pone.0060185 23533674 PMC3606337

[73] Hooks SB Callihan P Altman MK Hurst JH Ali MW Murph MM Regulators of G-Protein signaling RGS10 and RGS17 regulate chemoresistance in ovarian cancer cells. Mol Cancer (2010) 9:289 10.1186/1476-4598-9-289 21044322 PMC2988731

[74] Bonenfant D Towbin H Coulot M Schindler P Mueller DR van Oostrum J Analysis of dynamic changes in post-translational modifications of human histones during cell cycle by mass spectrometry. Mol Cell Proteomics (2007) 6(11):1917–32 10.1074/mcp.M700070-MCP200 17644761

[75] Garcia BA Mollah S Ueberheide BM Busby SA Muratore TL Shabanowitz J Chemical derivatization of histones for facilitated analysis by mass spectrometry. Nat Protoc (2007) 2(4):933–8 10.1038/nprot.2007.106 17446892 PMC4627699

[76] Plazas-Mayorca MD Zee BM Young NL Fingerman IM LeRoy G Briggs SD One-pot shotgun quantitative mass spectrometry characterization of histones. J Proteome Res (2009) 8(11):5367–74 10.1021/pr900777e 19764812 PMC2798817

[77] Sweet SM Li M Thomas PM Durbin KR Kelleher NL Kinetics of re-establishing H3K79 methylation marks in global human chromatin. J Biol Chem (2010) 285(43):32778–86 10.1074/jbc.M110.145094 20699226 PMC2963384

[78] Zee BM Levin RS Xu B LeRoy G Wingreen NS Garcia BA In vivo residue-specific histone methylation dynamics. J Biol Chem (2010) 285(5):3341–50 10.1074/jbc.M109.063784 19940157 PMC2823435

[79] Bartke T Borgel J DiMaggio PA Proteomics in epigenetics: new perspectives for cancer research. Brief Funct Genomics (2013) 12(3):205–18 10.1093/bfgp/elt002 23401080 PMC3662889

[80] Ho JW Bishop E Karchenko PV Nègre N White KP Park PJ ChIP-chip versus ChIP-seq: lessons for experimental design and data analysis. BMC Genomics (2011) 12:134 10.1186/1471-2164-12-134 21356108 PMC3053263

[81] Lakkaraju A Rodriguez-Boulan E Itinerant exosomes: emerging roles in cell and tissue polarity. Trends Cell Biol (2008) 18(5):199–209 10.1016/j.tcb.2008.03.002 18396047 PMC3754907

[82] Wittmann J Jack HM Serum microRNAs as powerful cancer biomarkers. Biochim Biophys Acta (2010) 1806(2):200–7 10.1016/j.bbcan.2010.07.002 20637263

[83] Benner SA Extracellular ‘communicator RNA.’ FEBS Lett (1988) 233(2):225–8 10.1016/0014-5793(88)80431-9 2454845

[84] Resnick KE Alder H Hagan JP Richardson DL Croce CM Cohn DE The detection of differentially expressed microRNAs from the serum of ovarian cancer patients using a novel real-time PCR platform. Gynecol Oncol (2009) 112(1):55–9 10.1016/j.ygyno.2008.08.036 18954897

[85] Zheng H Zhang L Zhao Y Yang D Song F Wen Y Plasma miRNAs as diagnostic and prognostic biomarkers for ovarian cancer. PLoS One (2013) 8(11):e77853 10.1371/journal.pone.0077853 24223734 PMC3815222

[86] Laios A O’Toole S Flavin R Martin C Kelly L Ring M Potential role of miR-9 and miR-223 in recurrent ovarian cancer. Mol Cancer (2008) 7:35 10.1186/1476-4598-7-35 18442408 PMC2383925

[87] Taylor DD Gercel-Taylor C MicroRNA signatures of tumor-derived exosomes as diagnostic biomarkers of ovarian cancer. Gynecol Oncol (2008) 110(1):13–21 10.1016/j.ygyno.2008.04.033 18589210

[88] Jacobs IJ Menon U Progress and challenges in screening for early detection of ovarian cancer. Mol Cell Proteomics (2004) 3(4):355–66 10.1074/mcp.R400006-MCP200 14764655

[89] Qu G Dubeau L Narayan A Yu MC Ehrlich M Satellite DNA hypomethylation vs. overall genomic hypomethylation in ovarian epithelial tumors of different malignant potential. Mutat Res (1999) 423(1–2):91–101 10.1016/S0027-5107(98)00229-2 10029684

[90] Ehrlich M Woods CB Yu MC Dubeau L Yang F Campan M Quantitative analysis of associations between DNA hypermethylation, hypomethylation, and DNMT RNA levels in ovarian tumors. Oncogene (2006) 25(18):2636–45 10.1038/sj.onc.1209145 16532039 PMC1449872

[91] Widschwendter M Jiang G Woods C Müller HM Fiegl H Goebel G DNA hypomethylation and ovarian cancer biology. Cancer Res (2004) 64(13):4472–80 10.1158/0008-5472.CAN-04-0238 15231656

[92] Ibanez de Caceres I Battagli C Esteller M Herman JG Dulaimi E Edelson MI Tumor cell-specific BRCA1 and RASSF1A hypermethylation in serum, plasma, and peritoneal fluid from ovarian cancer patients. Cancer Res (2004) 64(18):6476–81 10.1158/0008-5472.CAN-04-1529 15374957

[93] Wiley A Katsaros D Fracchioli S Yu H Methylation of the insulin-like growth factor binding protein-3 gene and prognosis of epithelial ovarian cancer. Int J Gynecol Cancer (2006) 16(1):210–8 10.1111/j.1525-1438.2006.00299.x 16445635

[94] Lyko F Brown R DNA methyltransferase inhibitors and the development of epigenetic cancer therapies. J Natl Cancer Inst (2005) 97(20):1498–506 10.1093/jnci/dji311 16234563

[95] Curt GA Kelley JA Fine RL Huguenin PN Roth JS Batist G A phase I and pharmacokinetic study of dihydro-5-azacytidine (NSC 264880). Cancer Res (1985) 45(7):3359–63 2408749

[96] Zambrano P Segura-Pacheco B Perez-Cardenas E Cetina L Revilla-Vazquez A Taja-Chayeb L A phase I study of hydralazine to demethylate and reactivate the expression of tumor suppressor genes. BMC Cancer (2005) 5:44 10.1186/1471-2407-5-44 15862127 PMC1131894

[97] Coronel J Cetina L Pacheco I Trejo-Becerril C González-Fierro A de la Cruz-Hernandez E A double-blind, placebo-controlled, randomized phase III trial of chemotherapy plus epigenetic therapy with hydralazine valproate for advanced cervical cancer. Preliminary results. Med Oncol (2011) 28(Suppl 1):S540–6 10.1007/s12032-010-9700-3 20931299

[98] Bast RC Jr. Mills GB Personalizing therapy for ovarian cancer: BRCAness and beyond. J Clin Oncol (2010) 28(22):3545–8 10.1200/JCO.2010.28.5791 20547987

[99] Watanabe Y Ueda H Etoh T Koike E Fujinami N Mitsuhashi A A change in promoter methylation of hMLH1 is a cause of acquired resistance to platinum-based chemotherapy in epithelial ovarian cancer. Anticancer Res (2007) 27(3B):1449–52 17595760

[100] Gifford G Paul J Vasey PA Kaye SB Brown R The acquisition of hMLH1 methylation in plasma DNA after chemotherapy predicts poor survival for ovarian cancer patients. Clin Cancer Res (2004) 10(13):4420–6 10.1158/1078-0432.CCR-03-0732 15240532

[101] Plumb JA Strathdee G Sludden J Kaye SB Brown R Reversal of drug resistance in human tumor xenografts by 2′-deoxy-5-azacytidine-induced demethylation of the hMLH1 gene promoter. Cancer Res (2000) 60(21):6039–44 11085525

[102] Dallol A Agathanggelou A Fenton SL Ahmed-Choudhury J Hesson L Vos MD RASSF1A interacts with microtubule-associated proteins and modulates microtubule dynamics. Cancer Res (2004) 64(12):4112–6 10.1158/0008-5472.CAN-04-0267 15205320

[103] El-Kalla M Onyskiw C Baksh S Functional importance of RASSF1A microtubule localization and polymorphisms. Oncogene (2010) 29(42):5729–40 10.1038/onc.2010.316 20697344

[104] Vos MD Martinez A Elam C Dallol A Taylor BJ Latif F A role for the RASSF1A tumor suppressor in the regulation of tubulin polymerization and genomic stability. Cancer Res (2004) 64(12):4244–50 10.1158/0008-5472.CAN-04-0339 15205337

[105] Shivakumar L Minna J Sakamaki T Pestell R White MA The RASSF1A tumor suppressor blocks cell cycle progression and inhibits cyclin D1 accumulation. Mol Cell Biol (2002) 22(12):4309–18 10.1128/MCB.22.12.4309-4318.2002 12024041 PMC133879

[106] Li Y Hu W Shen DY Kavanagh JJ Fu S Azacitidine enhances sensitivity of platinum-resistant ovarian cancer cells to carboplatin through induction of apoptosis. Am J Obstet Gynecol (2009) 200(2):e1–9 10.1016/j.ajog.2008.08.030 19110234

[107] Frost P Abbruzzese JL Hunt B Lee D Ellis M Synergistic cytotoxicity using 2′-deoxy-5-azacytidine and cisplatin or 4-hydroperoxycyclophosphamide with human tumor cells. Cancer Res (1990) 50(15):4572–7 1695122

[108] Kassler S Donninger H Birrer MJ Clark GJ RASSF1A and the taxol response in ovarian cancer. Mol Biol Int (2012) 2012:263267 10.1155/2012/263267 22548172 PMC3324163

[109] Fang F Balch C Schilder J Breen T Zhang S Shen C A phase 1 and pharmacodynamic study of decitabine in combination with carboplatin in patients with recurrent, platinum-resistant, epithelial ovarian cancer. Cancer (2010) 116(17):4043–53 10.1002/cncr.25204 20564122 PMC2930033

[110] Matei D Fang F Shen C Schilder J Arnold A Zeng Y Epigenetic resensitization to platinum in ovarian cancer. Cancer Res (2012) 72(9):2197–205 10.1158/0008-5472.CAN-11-3909 22549947 PMC3700422

[111] Joenje H Patel KJ The emerging genetic and molecular basis of Fanconi anaemia. Nat Rev Genet (2001) 2(6):446–57 10.1038/35076590 11389461

[112] Taniguchi T Tischkowitz M Ameziane N Hodgson SV Mathew CG Joenje H Disruption of the Fanconi anemia-BRCA pathway in cisplatin-sensitive ovarian tumors. Nat Med (2003) 9(5):568–74 10.1038/nm852 12692539

[113] Gupta A Godwin AK Vanderveer L Lu A Liu J Hypomethylation of the synuclein gamma gene CpG island promotes its aberrant expression in breast carcinoma and ovarian carcinoma. Cancer Res (2003) 63(3):664–73 12566312

[114] Kodama J Shinyo Y Hashen G Hongo A Yoshinouchi M Hiramatsu Y Heparanase messenger RNA expression in epithelial ovarian tumor. Int J Mol Med (2003) 12(6):961–4 10.3892/ijmm.12.6.961 14612974

[115] Shteper PJ Zcharia E Ashhab Y Peretz T Vlodavsky I Ben-Yehuda D Role of promoter methylation in regulation of the mammalian heparanase gene. Oncogene (2003) 22(49):7737–49 10.1038/sj.onc.1207056 14586400

[116] Choi JH Kwon HJ Yoon BI Kim JH Han SU Joo HJ Expression profile of histone deacetylase 1 in gastric cancer tissues. Jpn J Cancer Res (2001) 92(12):1300–4 10.1111/j.1349-7006.2001.tb02153.x 11749695 PMC5926683

[117] Zhu P Martin E Mengwasser J Schlag P Janssen KP Göttlicher M Induction of HDAC2 expression upon loss of APC in colorectal tumorigenesis. Cancer Cell (2004) 5(5):455–63 10.1016/S1535-6108(04)00114-X 15144953

[118] Khabele D Son DS Parl AK Goldberg GL Augenlicht LH Mariadason JM Drug-induced inactivation or gene silencing of class I histone deacetylases suppresses ovarian cancer cell growth: implications for therapy. Cancer Biol Ther (2007) 6(5):795–801 10.4161/cbt.6.5.4007 17387270

[119] Glaser KB Li J Staver MJ Wei RQ Albert DH Davidsen SK Role of class I and class II histone deacetylases in carcinoma cells using siRNA. Biochem Biophys Res Commun (2003) 310(2):529–36 10.1016/j.bbrc.2003.09.043 14521942

[120] Lee YS Lim KH Guo X Kawaguchi Y Gao Y Barrientos T The cytoplasmic deacetylase HDAC6 is required for efficient oncogenic tumorigenesis. Cancer Res (2008) 68(18):7561–9 10.1158/0008-5472.CAN-08-0188 18794144 PMC2978070

[121] Mulero-Navarro S Esteller M Epigenetic biomarkers for human cancer: the time is now. Crit Rev Oncol Hematol (2008) 68(1):1–11 10.1016/j.critrevonc.2008.03.001 18430583

[122] Chavez-Blanco A Segura-Pacheco B Perez-Cardenas E Taja-Chayeb L Cetina L Candelaria M Histone acetylation and histone deacetylase activity of magnesium valproate in tumor and peripheral blood of patients with cervical cancer. A phase I study. Mol Cancer (2005) 4(1):22 10.1186/1476-4598-4-22 16001982 PMC1198251

[123] Cincarova L Zdrahal Z Fajkus J New perspectives of valproic acid in clinical practice. Expert Opin Investig Drugs (2013) 22(12):1535–47 10.1517/13543784.2013.853037 24160174

[124] Kwiecinska P Wróbel A Taubøll E Gregoraszczuk EŁ Valproic acid, but not levetiracetam, selectively decreases HDAC7 and HDAC2 expression in human ovarian cancer cells. Toxicol Lett (2014) 224(2):225–32 10.1016/j.toxlet.2013.10.035 24200999

[125] Takai N Ueda T Nishida M Nasu K Narahara H A novel histone deacetylase inhibitor, Scriptaid, induces growth inhibition, cell cycle arrest and apoptosis in human endometrial cancer and ovarian cancer cells. Int J Mol Med (2006) 17(2):323–9 16391833

[126] Ueda T Takai N Nishida M Nasu K Narahara H Apicidin, a novel histone deacetylase inhibitor, has profound anti-growth activity in human endometrial and ovarian cancer cells. Int J Mol Med (2007) 19(2):301–8 10.3892/ijmm.19.2.301 17203205

[127] Marchion DC Bicaku E Daud AI Sullivan DM Munster PN Valproic acid alters chromatin structure by regulation of chromatin modulation proteins. Cancer Res (2005) 65(9):3815–22 10.1158/0008-5472.CAN-04-2478 15867379

[128] Bastian L Einsiedel HG Henze G Seeger K Shalapour S The sequence of application of methotrexate and histone deacetylase inhibitors determines either a synergistic or an antagonistic response in childhood acute lymphoblastic leukemia cells. Leukemia (2011) 25(2):359–61 10.1038/leu.2010.259 21072050

[129] Qian X LaRochelle WJ Ara G Wu F Petersen KD Thougaard A Activity of PXD101, a histone deacetylase inhibitor, in preclinical ovarian cancer studies. Mol Cancer Ther (2006) 5(8):2086–95 10.1158/1535-7163.MCT-06-0111 16928830

[130] Plumb JA Finn PW Williams RJ Bandara MJ Romero MR Watkins CJ Pharmacodynamic response and inhibition of growth of human tumor xenografts by the novel histone deacetylase inhibitor PXD101. Mol Cancer Ther (2003) 2(8):721–8 12939461

[131] Parker JP Nimir H Griffith DM Duff B Chubb AJ Brennan MP A novel platinum complex of the histone deacetylase inhibitor belinostat: rational design, development and in vitro cytotoxicity. J Inorg Biochem (2013) 124:70–7 10.1016/j.jinorgbio.2013.03.011 23603796

[132] Dizon DS Blessing JA Penson RT Drake RD Walker JL Johnston CM A phase II evaluation of belinostat and carboplatin in the treatment of recurrent or persistent platinum-resistant ovarian, fallopian tube, or primary peritoneal carcinoma: a Gynecologic Oncology Group study. Gynecol Oncol (2012) 125(2):367–71 10.1016/j.ygyno.2012.02.019 22366594 PMC3330705

[133] Dizon DS Damstrup L Finkler NJ Lassen U Celano P Glasspool R Phase II activity of belinostat (PXD-101), carboplatin, and paclitaxel in women with previously treated ovarian cancer. Int J Gynecol Cancer (2012) 22(6):979–86 10.1097/IGC.0b013e31825736fd 22694911

[134] Mackay HJ Hirte H Colgan T Covens A MacAlpine K Grenci P Phase II trial of the histone deacetylase inhibitor belinostat in women with platinum resistant epithelial ovarian cancer and micropapillary (LMP) ovarian tumours. Eur J Cancer (2010) 46(9):1573–9 10.1016/j.ejca.2010.02.047 20304628 PMC3244274

[135] Yang YT Balch C Kulp SK Mand MR Nephew KP Chen CS A rationally designed histone deacetylase inhibitor with distinct antitumor activity against ovarian cancer. Neoplasia (2009) 11(6):552–63 10.1593/neo.09204 19484144 PMC2685444

[136] Ebert MS Neilson JR Sharp PA MicroRNA sponges: competitive inhibitors of small RNAs in mammalian cells. Nat Methods (2007) 4(9):721–6 10.1038/nmeth1079 17694064 PMC3857099

[137] Elmén J Lindow M Schütz S Lawrence M Petri A Obad S LNA-mediated microRNA silencing in non-human primates. Nature (2008) 452(7189):896–9 10.1038/nature06783 18368051

[138] Gumireddy K Young DD Xiong X Hogenesch JB Huang Q Deiters A Small-molecule inhibitors of microrna miR-21 function. Angew Chem Int Ed Engl (2008) 47(39):7482–4 10.1002/anie.200801555 18712719 PMC3428715

[139] Xiao J Yang B Lin H Lu Y Luo X Wang Z Novel approaches for gene-specific interference via manipulating actions of microRNAs: examination on the pacemaker channel genes HCN2 and HCN4. J Cell Physiol (2007) 212(2):285–92 10.1002/jcp.21062 17516552

[140] Sorrentino A Liu CG Addario A Peschle C Scambia G Ferlini C Role of microRNAs in drug-resistant ovarian cancer cells. Gynecol Oncol (2008) 111(3):478–86 10.1016/j.ygyno.2008.08.017 18823650

[141] Jia W Eneh JO Ratnaparkhe S Altman MK Murph MM MicroRNA-30c-2* expressed in ovarian cancer cells suppresses growth factor-induced cellular proliferation and downregulates the oncogene BCL9. Mol Cancer Res (2011) 9(12):1732–45 10.1158/1541-7786.MCR-11-0245 22024689

[142] Cochrane DR Spoelstra NS Howe EN Nordeen SK Richer JK MicroRNA-200c mitigates invasiveness and restores sensitivity to microtubule-targeting chemotherapeutic agents. Mol Cancer Ther (2009) 8(5):1055–66 10.1158/1535-7163.MCT-08-1046 19435871 PMC4573391

[143] Leskelä S Leandro-García LJ Mendiola M Barriuso J Inglada-Pérez L Muñoz I The miR-200 family controls beta-tubulin III expression and is associated with paclitaxel-based treatment response and progression-free survival in ovarian cancer patients. Endocr Relat Cancer (2011) 18(1):85–95 10.1677/ERC-10-0148 21051560

[144] Yang N Kaur S Volinia S Greshock J Lassus H Hasegawa K MicroRNA microarray identifies Let-7i as a novel biomarker and therapeutic target in human epithelial ovarian cancer. Cancer Res (2008) 68(24):10307–14 10.1158/0008-5472.CAN-08-1954 19074899 PMC2762326

[145] Boyerinas B Park SM Murmann AE Gwin K Montag AG Zillhardt M Let-7 modulates acquired resistance of ovarian cancer to Taxanes via IMP-1-mediated stabilization of multidrug resistance 1. Int J Cancer (2012) 130(8):1787–97 10.1002/ijc.26190 21618519 PMC3230767

[146] Eitan R Kushnir M Lithwick-Yanai G David MB Hoshen M Glezerman M Tumor microRNA expression patterns associated with resistance to platinum based chemotherapy and survival in ovarian cancer patients. Gynecol Oncol (2009) 114(2):253–9 10.1016/j.ygyno.2009.04.024 19446316

[147] Lu L Schwartz P Scarampi L Rutherford T Canuto EM Yu H MicroRNA let-7a: a potential marker for selection of paclitaxel in ovarian cancer management. Gynecol Oncol (2011) 122(2):366–71 10.1016/j.ygyno.2011.04.033 21571355

[148] Tsang WP Kwok TT Let-7a microRNA suppresses therapeutics-induced cancer cell death by targeting caspase-3. Apoptosis (2008) 13(10):1215–22 10.1007/s10495-008-0256-z 18758960

[149] Yang C Cai J Wang Q Tang H Cao J Wu L Epigenetic silencing of miR-130b in ovarian cancer promotes the development of multidrug resistance by targeting colony-stimulating factor 1. Gynecol Oncol (2012) 124(2):325–34 10.1016/j.ygyno.2011.10.013 22005523

[150] van Jaarsveld MT Helleman J Boersma AW van Kuijk PF van Ijcken WF Despierre E miR-141 regulates KEAP1 and modulates cisplatin sensitivity in ovarian cancer cells. Oncogene (2013) 32(36):4284–93 10.1038/onc.2012.433 23045278

[151] Huh JH Kim TH Kim K Song JA Jung YJ Jeong JY Dysregulation of miR-106a and miR-591 confers paclitaxel resistance to ovarian cancer. Br J Cancer (2013) 109(2):452–61 10.1038/bjc.2013.305 23807165 PMC3721386

[152] Yu PN Yan MD Lai HC Huang RL Chou YC Lin WC Downregulation of miR-29 contributes to cisplatin resistance of ovarian cancer cells. Int J Cancer (2013) 134(3):542–51 10.1002/ijc.28399 23904094

[153] Guo Y Liao Y Jia C Ren J Wang J Li T MicroRNA-182 promotes tumor cell growth by targeting transcription elongation factor A-like 7 in endometrial carcinoma. Cell Physiol Biochem (2013) 32(3):581–90 10.1159/000354462 24021963

[154] Wang YQ Guo RD Guo RM Sheng W Yin LR MicroRNA-182 promotes cell growth, invasion, and chemoresistance by targeting programmed cell death 4 (PDCD4) in human ovarian carcinomas. J Cell Biochem (2013) 114(7):1464–73 10.1002/jcb.24488 23296900

[155] Snowdon J Zhang X Childs T Tron VA Feilotter H The microRNA-200 family is upregulated in endometrial carcinoma. PLoS One (2011) 6(8):e22828 10.1371/journal.pone.0022828 21897839 PMC3163579

[156] Ferlini C Raspaglio G Cicchillitti L Mozzetti S Prislei S Bartollino S Looking at drug resistance mechanisms for microtubule interacting drugs: does TUBB3 work? Curr Cancer Drug Targets (2007) 7(8):704–12 10.2174/156800907783220453 18220531

[157] Li Z Hu S Wang J Cai J Xiao L Yu L MiR-27a modulates MDR1/P-glycoprotein expression by targeting HIPK2 in human ovarian cancer cells. Gynecol Oncol (2010) 119(1):125–30 10.1016/j.ygyno.2010.06.004 20624637

[158] Zhu H Wu H Liu X Evans BR Medina DJ Liu CG Role of MicroRNA miR-27a and miR-451 in the regulation of MDR1/P-glycoprotein expression in human cancer cells. Biochem Pharmacol (2008) 76(5):582–8 10.1016/j.bcp.2008.06.007 18619946 PMC2628586

[159] Xie Z Cao L Zhang J miR-21 modulates paclitaxel sensitivity and hypoxia-inducible factor-1alpha expression in human ovarian cancer cells. Oncol Lett (2013) 6(3):795–800 10.3892/ol.2013.1432 24137413 PMC3789026

[160] Marks JR Davidoff AM Kerns BJ Humphrey PA Pence JC Dodge RK Overexpression and mutation of p53 in epithelial ovarian cancer. Cancer Res (1991) 51(11):2979–84 2032235

[161] Weinberg RA The Biology of Cancer: Second Edition. New York, NY: Garland Science (2014). 876 p.

[162] Ciucci A De StefanoI Vellone VG Lisi L Bottoni C Scambia G Expression of the glioma-associated oncogene homolog 1 (gli1) in advanced serous ovarian cancer is associated with unfavorable overall survival. PLoS One (2013) 8(3):e60145 10.1371/journal.pone.0060145 23555905 PMC3610749

[163] Sen M Thomas SM Kim S Yeh JI Ferris RL Johnson JT First-in-human trial of a STAT3 decoy oligonucleotide in head and neck tumors: implications for cancer therapy. Cancer Discov (2012) 2(8):694–705 10.1158/2159-8290.CD-12-0191 22719020 PMC3668699

[164] Raskatov JA Meier JL Puckett JW Yang F Ramakrishnan P Dervan PB Modulation of NF-kappaB-dependent gene transcription using programmable DNA minor groove binders. Proc Natl Acad Sci U S A (2012) 109(4):1023–8 10.1073/pnas.1118506109 22203967 PMC3268328

[165] Moellering RE Cornejo M Davis TN Del BiancoC Aster JC Blacklow SC Direct inhibition of the NOTCH transcription factor complex. Nature (2009) 462(7270):182–8 10.1038/nature08543 19907488 PMC2951323

[166] Liu M Wang F Wen Z Shi M Zhang H Blockage of STAT3 signaling pathway with a decoy oligodeoxynucleotide inhibits growth of human ovarian cancer cells. Cancer Invest (2014) 32(1):8–12 10.3109/07357907.2013.861471 24328557

[167] Ataie-Kachoie P Badar S Morris DL Pourgholami MH Minocycline targets the NF-kappaB Nexus through suppression of TGF-beta1-TAK1-IkappaB signaling in ovarian cancer. Mol Cancer Res (2013) 11(10):1279–91 10.1158/1541-7786.MCR-13-0239 23858099

[168] Ang JE Gourley C Powell CB High H Shapira-Frommer R Castonguay V Efficacy of chemotherapy in BRCA1/2 mutation carrier ovarian cancer in the setting of PARP inhibitor resistance: a multi-institutional study. Clin Cancer Res (2013) 19(19):5485–93 10.1158/1078-0432.CCR-13-1262 23922302

